# Potential biomarkers and therapeutic targets of idiopathic pulmonary arterial hypertension

**DOI:** 10.14814/phy2.15101

**Published:** 2022-01-04

**Authors:** Wenjun He, Xi Su, Lingdan Chen, Chunli Liu, Wenju Lu, Tao Wang, Jian Wang

**Affiliations:** ^1^ State Key Laboratory of Respiratory Diseases Guangdong Key Laboratory of Vascular Diseases National Clinical Research Center for Respiratory Diseases Guangzhou Institute of Respiratory Health The First Affiliated Hospital of Guangzhou Medical University Guangzhou China; ^2^ Department of Pulmonary Medicine Amsterdam University Medical Center Location VU University Medical Center Amsterdam The Netherlands; ^3^ Shanghai Chest Hospital, Shanghai Jiao Tong University Shanghai China; ^4^ Division of Cardiology Department of Medicine University of California San Diego California USA

**Keywords:** biomarker, gene analysis, GEO, IPAH

## Abstract

**Background:**

Peripheral blood mononuclear cells (PBMCs) play an important role in the pathogenesis of pulmonary arterial hypertension (PAH). However, the specific roles of PBMCs in the development and progression of idiopathic PAH (IPAH) have not been fully understood.

**Methods:**

Here, differentially expressed genes (DEGs) of PBMCs or lung tissues between IPAH patients and healthy controls were identified via bioinformatics analysis of Gene Expression Omnibus (GEO) datasets GSE33463 and GSE48149, respectively. Subsequently, extensive target prediction and network analysis were performed to assess protein–protein interaction (PPI) networks, Gene Ontology (GO) terms, and pathway enrichment for DEGs. Co‐expressed DEGs between PBMCs and lung tissues coupled with corresponding predicted miRNAs involved in PAH were also assessed. We identified 251 DEGs in PBMCs and 151 DEGs in lung tissue samples from IPAH. PDK4, RBPMS2, and PDE5A expression were altered in both PBMCs and lung tissues from IPAH patients compared to healthy control.

**Results:**

CXCL8, JUN, TLR8, IL1B, and TLR7 could be implicated as the hub genes in PBMCs, whereas ENO1, STAT1, CXCL10, GPI, and IRF1 in lung tissues. Finally, co‐expressed DEGs of PDK4, RBPMS2, and PDE5A coupled with corresponding predicted miRNAs, especially miR‐103a‐3p, miR‐185‐5p, and miR‐515‐5p, are significantly associated with IPAH.

**Conclusion:**

Our findings collectively suggest that the expression levels of PDK4, RBPMS2, and PDE5A in PBMCs are associated with the expression of these genes in lung tissues. Thus, these molecules may serve as potential circulating biomarkers and/or possible therapeutic targets for IPAH.

## Graphical abstract

It is reported that PBMCs play a vital role in the pathogenesis of pulmonary arterial hypertension. An analysis of gene expression in lung homogenates may provide insight into genes involved in vascular remodeling in IPAH patients. We thus wrote this letter to discuss the necessity to identify the potential biomarkers and possible therapeutic targets for IPAH by a bioinformatics analysis of the transcriptome in PBMCs and lung tissues of IPAH.

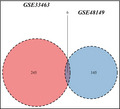

## 1 INTRODUCTION

Idiopathic Pulmonary Arterial Hypertension (IPAH) is a rare and progressive disease with a poor prognosis, characterized by a progressive increase of pulmonary artery (PA) pressure and pulmonary vascular resistance (PVR) (Keating, 2016; Lau et al., 2017; Maron & Diagnosis, 2016). Although the mechanisms of IPAH remain unknown, there is growing evidence indicating that peripheral blood mononuclear cells (PBMCs) contribute to the pathogenesis of IPAH (Cheadle et al., 2012; Hoffmann et al., 2016; Risbano et al., 2010; Sarrion et al., 2015). Circulating PBMCs include lymphocytes, monocytes, stem cells, and other cell types. Compared to lung tissues, PBMCs can be collected from a broader range of patients; thus, PBMCs are used to study biomarkers for various diseases (Baine et al., 2012; Castaldo et al., 2019; Goleva et al., 2019; Guo et al., 2019; Yang et al., 2016). Lung tissues contain vessels and provide the microenvironment for pulmonary vascular cells, which are pathologically changed in pulmonary hypertension.

The gene expression analysis in lung homogenates may provide insight into genes involved in vascular remodeling in IPAH patients (Hoffmann et al., 2016; Yuan et al., 2016). However, lung tissues are not available from most of IPAH patients. Numerous studies suggest that PBMCs infiltrate lung tissues and play an important role in the pathogenesis of PH (Cheadle et al., 2012; Chesne et al., 2014; Risbano et al., 2010; Sarrion et al., 2015). Elevated numbers of inflammatory cells have been observed in the lungs of patients with IPAH. The recruited inflammatory cells can release mediators to directly change the vascular microenvironment, promoting the proliferation of pulmonary smooth muscle cells and recruiting more circulating inflammatory cells, further aggravating the progress of the disease (Hassoun et al., 2009; Hoffmann et al., 2011; Marsh et al., 2018; Perros et al., 2007; Pienn et al., 2018; Savai et al., 2012). PBMCs play a critical role in activating the innate immune response, which can migrate into the lung tissue (Cheadle et al., 2012; Foris et al., 2016; Khan & Kaihara, 2019; Lenna et al., 2013; Yamamoto, 2011; Yeager et al., 2012). However, the mechanism of how circulating inflammatory cells is recruited to lung tissues and what relations between PBMCs and lung tissues are unknown. To our knowledge, high‐throughput technologies such as microarray expression profiles of PBMCs and lung tissues have provided powerful weapons for the diagnosis and prognosis of IPAH patients. Therefore, to make full use of expression profiles data of PBMCs and lung tissues from IPAH patients and better understand the pathogenesis of IPAH, we utilized bioinformatics analysis to explore hub genes and the critical mechanism of IPAH.

Here, we hypothesized that transcriptome changes between PBMCs and lung tissues contribute to the recruitment of inflammation cells, the changes of the vascular microenvironment, and pulmonary vascular remodeling. Thus, in this study, we conducted a bioinformatics analysis for the transcriptome in PBMCs and lung tissues between IPAH patients and healthy control to investigate the contribution of recruited immune cells in lung tissues to the development of IPAH with the hope of identifying potential biomarkers and therapeutic targets for IPAH.

## 2 METHODS

### 2.1 Sources of the gene data

**FIGURE 1:**
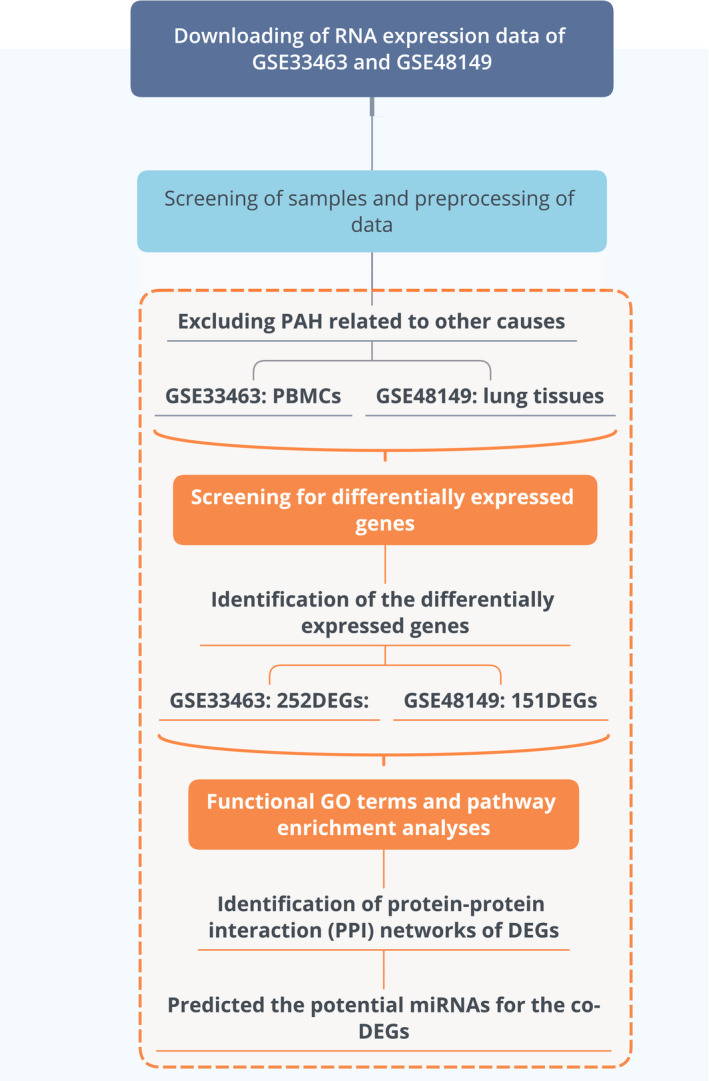
Workflow of this study. DEGs: differentially expressed genes; IPAH: idiopathic pulmonary arterial hypertension; PBMCs: peripheral blood mononuclear cells

GSE33463 and GSE48149 datasets were downloaded from Gene Expression Omnibus (GEO) database (http://www.ncbi.nlm.nih.gov/geo/), and the detection platforms for both GSE33463 and GSE48149 datasets were Illumina human V3.0 Expression BeadChip (Edgar et al., 2002). The gene expression profiles of PBMCs from 30 IPAH patients were compared to 41 healthy volunteers. The gene expression profiles of lung tissues are available from 17 donors who underwent lung transplantation (Table 1). These previous studies can provide insightful viewpoints concerning the epidemiological characteristics and phenotyping data related to IPAH (Cheadle et al., 2012; Hsu et al., 2011). They were both used to explore the differentially expressed genes between IPAH patients and healthy controls. The workflow of this study is illustrated in Figure 1.

**TABLE 1:** Demographic and clinical characteristics of the patients with IPAH

Variable	PBMC		Lung tissue	
	Control (n = 41)	IPAH (n = 30)	Control (n = 9)	IPAH (n = 8)
Age, (years)	45	52	53.0	35.7
Gender, n (%)				
Male	7 (9.9)	5 (7.0)	4 (23.5)	2 (11.8)
Female	34 (47.9)	25 (35.2)	5 (29.4)	6 (35.3)
Hemodynamic and pulmonary function parameters (Mean±SD)				
mPAP (mmHg)	N/A	66.1 ± 9.1	N/A	60.0 ± 13.3
RA (mmHg)	N/A	7.7 ± 4.2 (0)	N/A	N/A
CI (%)	N/A	2.61 ± 0.69 (0)	N/A	N/A
FVC %/DLCO %	N/A	N/A	N/A	2.02 ± 1.9

Note

N/A: not available; mPAP, mean Pulmonary Artery Pressure; RA, Right Atrium; CI, Cardiac Index; FVC, Forced Vital Capacity; DLCO, Diffusion Capacity for Carbon Monoxide.

### 2.2 Identification of the differentially expressed genes

R packages of “Illumina” and “limma” (http://www.bioconductor.org/packages/release/bioc/html/limma.html), provided by a Bioconductor project, were applied to assess GSE33463 and GSE48149 RAW datasets. We used background correction, quantile normalization, probe summarization, and log2‐transformation to create a robust multi‐array average (RMA), a log‐transformed perfect match, and a mismatch probe (PM and MM) methods. The Benjamin–Hochberg method was used to adjust original p‐values, and the false discovery rate (FDR) procedure was used to calculate fold‐changes (FC). Gene expression values of 2 ^|log2 FC|^ >2 and adjusted *p *< 0.05 were used for DEGs of PBMCs; 2^|log2 FC|^ >1.5 and adjusted *p *< 0.05 were used to identify DEGs of lung tissues. Then, two Volcano plots were adopted to visualize the expression differences by R 3.5.0. Additionally, we calculated and made Venn diagrams for co‐DEGs for the two datasets.

### 2.3 Functional analysis

The PBMCs‐DEGs and lung‐DEGs were subjected for functional annotation and Gene Ontology/ Kyoto Encyclopedia of Genes and Genomes (GO/KEGG) enrichment analysis for annotation, visualization, and integrated discovery of bioinformatics resources by DAVID (http://david.abcc.ncifcrf.gov/). GO terms and KEGG maps of biological functions associated with a *p *< 0.05 were significantly enriched. Besides, we presented different bio‐functions of PBMC‐DEGs and lung‐DEGs in biological processes, molecular functions, and cellular components from DAVID, respectively.

### 2.4 Identification of protein–protein interaction (PPI) networks of DEGs

PPI networks of PBMCs‐DEGs and lung‐DEGs were analyzed using the search tool for retrieving interacting genes (STRING database, V10.5; http://string‐db.org/) that can predict protein functional associations and protein–protein interactions. Subsequently, Cytoscape software (V3.5.1; http://cytoscape.org/) was applied to analyze biological networks and node degrees after downloading the analytic results of the STRING database.

### 2.5 Prediction of potential miRNAs for the co‐DEGs

Finally, we applied online prediction tools utilizing mirDIP (http://ophid.utoronto.ca/mirDIP), miRDB (http://mirdb.org/), and mirWalk (http://mirwalk.umm.uni‐heidelberg.de/) to predict potential miRNAs could target the co‐DEGs in lung tissue and PBMCs, respectively. We then made a Venn diagram for the common potential miRNAs of the mirDIP, miRDB, and miRWalk.

## 3 RESULTS IDENTIFICATION OF DEGS

**FIGURE 2:**
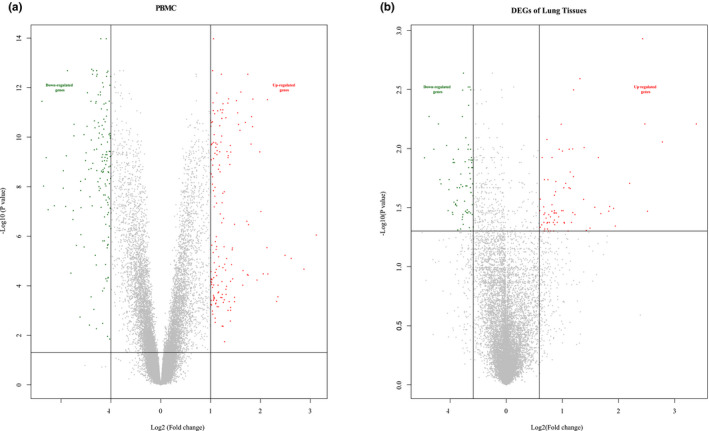
Volcano plots of DEGs. a.results of volcano plot for DEGs expression related to PBMCs in PAH. b. results of the volcano plot for DEGs expression related to lung tissues in PAH. Red, upregulated expression. Green, downregulated expression

**FIGURE 3:**
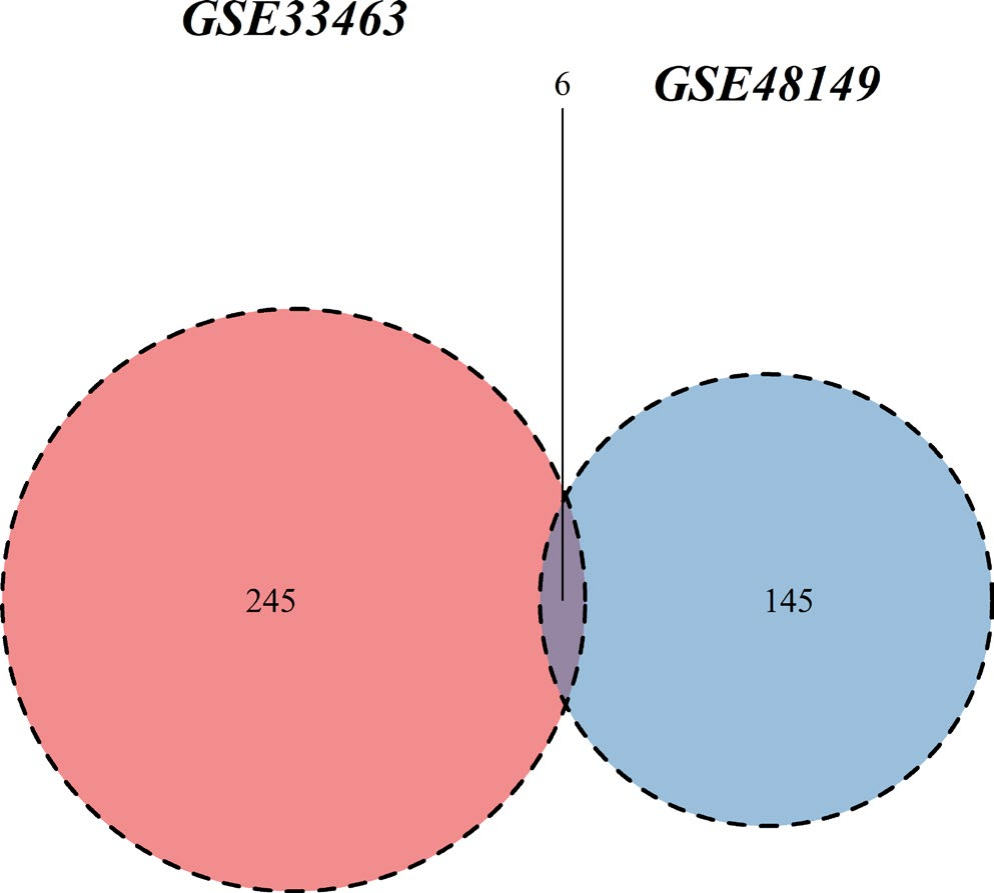
Venn diagrams of DEGs. There are 251 DEGs related to PBMCs and 151 DEGs related to the lung tissues in PAH patients. Gray, 6 co‐expressed DEGs were identified based on the DEG overlaps between the two datasets; Red, differentially expressed genes of PBMCs in PAH; Blue, differentially expressed genes of lung tissue in PAH

As indicated in Figure 2a, we found 251 DEGs in PBMCs of IPAH patients compared to healthy controls, including 110 downregulated genes and 141 upregulated genes. We found 151 DEGs in IPAH patients compared with the control population, including 73 downregulated and 78 upregulated genes in the lung tissues. The Volcano plots in Figure 2b illustrated the expression differences. And we identified six co‐expressed DEGs based on the DEG overlaps between the two datasets (Figure 3). As indicated in Table 2, PDK4, RBPMS2, and PDE5A were upregulated in both PBMC and lung tissues from IPAH patients compared to healthy control. TNFAIP3, CCL3, and PHLDA1 were found to be upregulated in lung tissue but downregulated in PBMCs.

**TABLE 2:** co‐DEGs of PBMCs and lung tissues

Gene	logFC.PBMCs	logFC. Lung tissues
TNFAIP3	−2.383422913	2.467540092
PDK4	1.479404085	2.194679977
RBPMS2	1.249352733	0.741858209
CCL3	−1.167271751	1.941109376
PHLDA1	−1.05684707	1.311408233
PDE5A	1.005595159	0.7671995

Note

Red genes: co‐upregulated genes in PBMCs and lung tissues; Black genes: inconsistently regulated genes between PBMCs and lung tissues.

### 3.1 Functional GO terms and pathway enrichment analyses

The functional GO terms enrichment was performed to reveal IPAH‐related GO categories such as biological processes, molecular functions, cellular components, and KEGG. For biological processes, we identified that the common between PBMCs with the lung tissues is apoptotic progress. For the cellular components, we found it were cytosol and mitochondrion. Their differentially expressed genes have common molecular functions in protein binding, protein homodimerization activity.

The top 10 GO terms of PBMCs‐IPAH are shown in Table 3. For DEGs in PBMCs‐IPAH, the most enriched GO terms of biological processes were associated with oxygen transport, blood coagulation, and apoptotic process. In the cellular component category, enriched GO terms were mainly associated with hemoglobin complex, platelet alpha granule membrane, and mitochondrion. Besides, GO terms enriched for DEGs in PBMCs‐IPAH included oxygen transport activity, protein binding, and oxygen binding in the molecular function category. To explore the significant enrichment of DEGs of PBMCs‐IPAH in pathway terms, we performed pathway annotation of DEGs and obtained DEGs involved in all pathway terms. The DEGs of PBMCs‐IPAH were enriched mainly in cytokine–cytokine receptor interaction, Toll‐like receptor signaling pathway, NF‐kappa B signaling pathway, and the chemokine signaling pathway. It is reported that oxygen transport activity plays a vital role in the vascular tone of pulmonary artery smooth muscle cells and hypoxia stimulates vasoconstriction of the pulmonary vascular (Wu et al., 2021). Cytokine–cytokine receptor interaction, chemokine signaling pathway, and Toll‐like receptor signaling pathway are known drivers to pulmonary vascular remodeling and established PH (Farkas et al., 2019; Xiao et al., 2020). NF‐kappa B signaling pathway has been reported as a therapeutic target for PH (Liu et al., 2020; Shi et al., 2018; Zhang et al., 2019; Zuo et al., 2020).

**TABLE 3:** Gene ontology (GO) and pathway enrichment analysis of PBMCs‐DEGs

Category	ID	Term	Count	*p* value
BP	GO:0015671	Oxygen transport	6	9.09E−07
	GO:0007596	Blood coagulation	13	4.75E−06
	GO:0051607	Defense response to virus	12	9.64E−06
	GO:0006915	Apoptotic process	22	1.42E−05
	GO:0070098	Chemokine‐mediated signaling pathway	8	3.50E−05
	GO:2001240	Negative regulation of extrinsic apoptotic signaling Pathway in absence of ligand	6	1.05E−04
	GO:0006954	Inflammatory response	16	1.20E−04
	GO:0032757	Positive regulation of interleukin−8 production	5	3.19E−04
	GO:0010941	Regulation of cell death	4	3.21E−04
	GO:0001774	Microglial cell activation	4	4.24E−04
CC	GO:0005833	Hemoglobin complex	7	3.20E−09
	GO:0031092	Platelet alpha granule membrane	4	5.10E−04
	GO:0005829	Cytosol	63	5.47E−04
	GO:0072562	Blood microparticle	8	0.003133417
	GO:0000228	Nuclear chromosome	5	0.004393052
	GO:0000786	Nucleosome	6	0.006657139
	GO:0000788	Nuclear nucleosome	4	0.01776436
	GO:0016234	Inclusion body	3	0.021071293
	GO:1990622	CHOP‐ATF3 complex	2	0.024974475
	GO:0005739	Mitochondrion	26	0.028572338
MF	GO:0005344	Oxygen transporter activity	6	6.25E−07
	GO:0005515	Protein binding	149	1.28E−06
	GO:0019825	Oxygen binding	6	3.38E−04
	GO:0046982	Protein heterodimerization activity	16	0.001070882
	GO:0042803	Protein homodimerization activity	21	0.001278347
	GO:0043565	Sequence‐specific DNA binding	16	0.003068635
	GO:0000982	Transcription factor activity, RNA polymerase II core Promoter proximal region sequence‐specific bin	4	0.003108637
	GO:0008009	Chemokine activity	5	0.003638514
	GO:0003924	GTPase activity	9	0.011222782
	GO:0008134	Transcription factor binding	10	0.011708629
KEGG	hsa05164	Influenza A	13	2.75E−05
	hsa04060	Cytokine–cytokine receptor interaction	13	6.49E−04
	hsa04380	Osteoclast differentiation	9	0.001415882
	hsa05202	Transcriptional misregulation in cancer	10	0.001726853
	hsa04620	Toll‐like receptor signaling pathway	8	0.001801965
	hsa05134	Legionellosis	6	0.001895755
	hsa04064	NF‐kappa B signaling pathway	7	0.003082372
	hsa04062	Chemokine signaling pathway	10	0.003588139
	hsa05162	Measles	8	0.006385733
	hsa05160	Hepatitis C	8	0.006385733

The top 10 GO terms of lung‐IPAH were performed and shown in Table 4. For DEGs in lung‐IPAH, the most enriched GO terms of the biological process were associated with an apoptotic process, erythrocyte differentiation, DNA metabolic processes, regulation of growth, and metabolic process. In the cellular component category, enriched GO terms were mainly associated with hemoglobin complex, platelet alpha granule membrane, and cytosol. Furthermore, in the molecular function category, GO terms enriched for DEGs in lung‐IPAH included metabolic pathways, biosynthesis of amino acids, lysosome, and carbon metabolism. To explore the significant enrichment of DEGs of lung‐IPAH in pathway terms, we performed pathway annotation of DEGs and obtained DEGs involved in all pathway terms in Table 4. The DEGs of lung‐IPAH were enriched mainly with metabolic pathways, biosynthesis of antibiotics, biosynthesis of amino acids, and lysosome (Table 4). A recent 4‐month, open‐label study also showed that decreasing PDH(a metabolic‐related molecular) has reduced mean PAP and PVR and improved functional capacity in IPAH patients with SIRT3 and UCP2 variants (Spiekerkoetter et al.,). Metabolic abnormalities cause PAH or are secondary to pulmonary vascular disease and subsequent RV failure (Paulin & Michelakis, 2014).

**TABLE 4:** Gene ontology (GO) and pathway enrichment analysis of lung‐DEGs

Category	ID	Term	Count	*p* value
BP	GO:0006915	Apoptotic process	22	0.009726238
	GO:0030218	Erythrocyte differentiation	5	0.009950815
	GO:0016032	Viral process	14	0.011693079
	GO:0006853	Carnitine shuttle	3	0.014621594
	GO:0016192	Vesicle‐mediated transport	9	0.015859207
	GO:0006259	DNA metabolic process	4	0.017119889
	GO:0040008	Regulation of growth	5	0.025761733
	GO:0008152	Metabolic process	9	0.0270116
	GO:0006886	Intracellular protein transport	11	0.029445951
	GO:0006183	GTP biosynthetic process	3	0.029961661
CC	GO:0070062	Extracellular exosome	99	1.70E−08
	GO:0005739	Mitochondrion	53	3.35E−06
	GO:0005730	Nucleolus	35	1.42E−04
	GO:0005829	Cytosol	91	0.001448734
	GO:0005743	Mitochondrial inner membrane	20	0.001658054
	GO:0030131	Clathrin adaptor complex	4	0.003761553
	GO:0000139	Golgi membrane	23	0.004638949
	GO:0015629	Actin cytoskeleton	12	0.004950301
	GO:0005789	Endoplasmic reticulum membrane	30	0.005118336
	GO:0005840	Ribosome	10	0.00669396
MF	GO:0005515	Protein binding	219	7.77E−05
	GO:0003824	Catalytic activity	11	0.006071153
	GO:0004843	Thiol‐dependent ubiquitin‐specific protease activity	7	0.006401087
	GO:0004527	Exonuclease activity	4	0.006744449
	GO:0042803	Protein homodimerization activity	26	0.010286664
	GO:0098641	Cadherin binding involved in cell–cell adhesion	13	0.018626355
	GO:0008135	Translation factor activity, RNA binding	4	0.021835325
	GO:0008237	Metallopeptidase activity	6	0.026779975
	GO:0015078	Hydrogen ion transmembrane transporter activity	4	0.028323568
	GO:0030170	Pyridoxal phosphate binding	5	0.030629693
KEGG	hsa01100	Metabolic pathways	49	9.53E−04
	hsa01130	Biosynthesis of antibiotics	15	0.001017114
	hsa01230	Biosynthesis of amino acids	7	0.009966251
	hsa04142	Lysosome	9	0.012113099
	hsa05100	Bacterial invasion of epithelial cells	7	0.014459025
	hsa01200	Carbon metabolism	8	0.025410887
	hsa00230	Purine metabolism	10	0.036272174
	hsa04145	Phagosome	9	0.038150082

### 3.2 PPI network analysis

**FIGURE 4:**
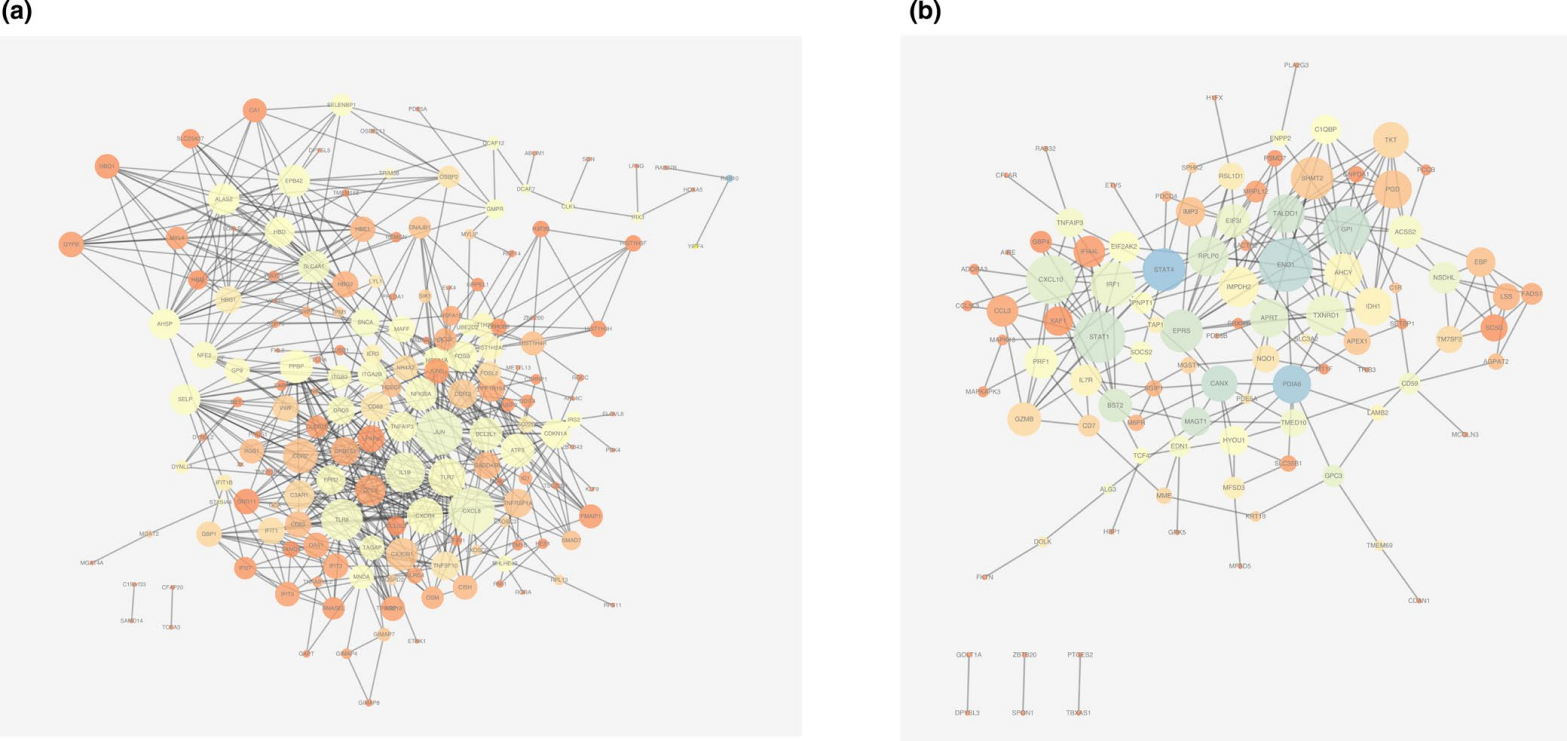
PPI network. PPI networks from a and b were constructed using the STRING database for DEGs related to PBMCs and lung tissues, respectively (threshold>0.5). a. CXCL8, JUN, and TLR8 could be implicated as the hub genes in PBMCs. b.ENO1, STAT1, and CXCL10 could be implicated as the hub genes in lung tissues

We identified 176 and 103 nodes from the PPI network of PBMC‐ and lung tissue‐ IPAH DEGs, respectively (Figure 4a,b). Here, the hub nodes, CXCL8(degree38), JUN (degree37), TLR8(degree31), IL1B(degree29), and TLR7(degree26), are demonstrated with a relatively higher degree in PBMC (GSE33463). On the other side, hub genes of ENO1(degree17), STAT1(degree16), CXCL10(degree15), GPI (degree14), and IRF1(degree13) are associated as hub genes in related to lung tissue‐IPAH.

### 3.3 Predicted miRNAs of Co‐DEGs

The potential miRNAs targeting the co‐DEGs were predicted by mirDIP, miRDB, and mirWalk. We plotted a Venn diagram to identify the potential miRNAs for PDK4, RBPMS2, CCL3, and PDE5A, respectively (Table 5). The corresponding predicted miRNA coupled with PDK4 were hsa‐miR‐103a‐3p and hsa‐miR‐148a‐3p. The predicted miRNA targeted with RBPMS2 were hsa‐miR‐185‐5p and hsa‐miR‐92, and PDE5A is coupled with hsa‐miR‐515‐5p, hsa‐miR‐507, hsa‐miR‐19b‐3p, hsa‐miR‐382‐5p, and hsa‐miR‐300.

**TABLE 5:** The Genomes (KEGG) pathways enrichment among predicted miRNAs and co‐DEGs

Genes	Predicted miRNAs	pathway	*p* value
PDK4	hsa‐miR−103a−3p	Regulation of pyruvate dehydrogenase (PDH) complex	1.09E−02
	hsa‐miR−148a−3p	Pyruvate metabolism	2.00E−02
		Signaling by Retinoic Acid	2.51E−02
		Pyruvate metabolism and Citric Acid (TCA) cycle	3.41E−02
		The citric acid (TCA) cycle and respiratory electron transport	7.82E−02
		Signaling by Nuclear Receptors	1.07E−01
		Signal Transduction	7.23E−01
		Metabolism	7.73E−01
RBPMS2	hsa‐miR−185‐5p	–	–
	hsa‐miR−920	–	–
PDE5A	hsa‐miR−515‐5p	cGMP effects	6.33E−03
	hsa‐miR−507	Nitric oxide stimulates guanylate cyclase	1.44E−02
	hsa‐miR−19b−3p	Platelet homeostasis	4.27E−02
	hsa‐miR−382‐5p	Hemostasis	2.58E−01
	hsa‐miR−300		

## 4 DISCUSSION

IPAH is a progressive disorder characterized by progressive pulmonary arterial narrowing (Keating, 2016; Lau et al., 2017; Maron & Diagnosis, 2016). Although the pathogenesis of IPAH remains still unclear, increasing lines of evidence suggest that PBMCs may be involved in the pathogenesis of IPAH. It has been reported that PBMCs changes would be reflected by specific transcriptional changes in these cells in PAH patients (Cheadle et al., 2012; Hoffmann et al., 2016; Sarrion et al., 2015).

Endothelial progenitor cells, which belong to PBMCs, have been accumulating in the lung upon PH‐induction (Yan et al., 2016). Moreover, bone marrow transplantation from PH mice to healthy mice increased the PA pressure in healthy mice (Levy et al., 2019; Nikolic & Yu, 2016). These may suggests that the changes in the PBMC genes might be the cause and effect of IPAH. Thus, we sought to explore the possible association between PBMCs and lung tissues in IPAH with the hope of identifying potential biomarkers or novel therapeutic targets for IPAH.

For DEGs in PBMCs‐IPAH, the most enriched GO terms of biological processes and molecular functions were associated with the oxygen transport activity and oxygen binding. Given the fact that the lung is the major organ for oxygen transport, our findings might suggest a possible relationship between PBMCs and lung tissues concerning oxygen transportation. Some studies have shown that in IPAH patients, due to increased PVR, the right ventricle's compensation is not enough (decreased cardiac output) to meet the demand for oxygen transport, resulting in inefficient lung gas exchange, and low perfusion (Tang et al., 2017).

Apart from the genes that are not consistent with upregulation and downregulation, there still remain four genes altered in both PBMCs and lung tissues from IPAH patients compared to healthy controls, including PDK4, RBPMS2, and PDE5A, which are all upregulated in IPAH patients.

Among these genes, PDE5A has been used as a target for IPAH therapy, including drug such as sildenafil, tadalafil, vardendafil, and so on. PDE5A is a phosphodiesterase specifically binding to cGMP, which is the main enzyme that metabolizes cGMP. Previous studies on PH animal models and PASMC from IPAH patients have shown that PDE5A participates in the excessive proliferation of PASMC. Wharton et al. demonstrated that PDE5A inhibition decreases DNA synthesis via increasing cellular cGMP level and stimulated the apoptosis of human PASMC (Lan et al., 2018; Osinski et al., 2001; Rai et al., 2018; Wharton et al., 2005).

PDK4, a gene coding for an enzyme that suppresses the mitochondrial activity in favor of glycolysis. Yuan K et al. found that increased PDK4 is associated with PAH pericyte hyperproliferation and reduced endothelial‐pericyte interactions (Yuan et al., 2016). Piao et al. showed that pyruvate dehydrogenase kinase (PDK) is activated in the right ventricular hypertrophy (RVH), causing an increase in glycolysis relative to glucose oxidation that impairs the right ventricular function (Piao et al., 2013). However, another DEG, RBPMS2, to the best of our knowledge, has not been reported to be associated with IPAH. Several studies have shown that RBPMS2 is related to the plasticity of the smooth muscle cells. Akerberg et al. have demonstrated that the proteins encoded by RBPMS2 may play a key role in regulating the cardiomyocyte differentiation, proliferation, survival, and/ or contractility (Kaufman et al., 2018). Sagnol et al. demonstrated that RBPMS2 is related to stomach smooth muscle development and plasticity. The excessive proliferation of vascular smooth muscle is one of the main pathological changes of PH (Sagnol et al., 2016), the relevant mechanisms of RBPMS2 in PAH development need to be further explored.

These findings suggest that the expression of PDK4, RBPMS2, and PDE5A in PBMCs could predict the expression levels of these genes in lung tissues and might serve as circulating potential biomarkers and/or therapeutic targets for IPAH patients. Further evaluation is ongoing in our group to provide more solid evidence to determine whether these DEGs could serve as biomarkers and/or therapeutic targets for IPAH.

## 5 CONCLUSION

These findings demonstrate the expression of PDK4, RBPMS2, and PDE5A in PBMCs could predict the expression levels of these genes in lung tissue and might serve as circulating biomarkers for IPAH.

### 5.1 Limitation

The clinical characteristics of patients were collected from the GEO database. The fundamental limitation is the lack of ethnic or geographic diversity in our samples, which may affect the reliability of the results. In addition, since the GEO dataset of PBMCs or lung tissues of IPAH are very limited, and the datasets belonging to the same probe‐type are even less, we selected two datasets with the largest sample size to analyze the differentially expressed genes. Further studies with larger sample sizes, more diverse ethnicities, and less heterogeneity are warranted to test our hypothesis.

## CONFLICT OF INTEREST

No potential conflict of interest was disclosed.

## AUTHOR CONTRIBUTION

Study Design: Wenjun He, Xi Su, Tao Wang, and Jian Wang. Data Analysis: Wenjun He, Xi Su, Lingdan Chen, and Chunli Liu. Manuscript writing: Wenjun He, Xi Su, and Wenju Lu. All authors approved the final version of the manuscript, agree to be accountable for all aspects of the work in ensuring that questions related to the accuracy or integrity of any part of the work are appropriately investigated and resolved. All persons designated as authors qualify for authorship, and all those who qualify for authorship are listed. All authors contributed to data interpretation, manuscript writing, and critical analysis of the manuscript; and provided final approval for manuscript submission.

## GUARANTOR

Jian Wang and Tao Wang.

## Data Availability

Publicly available datasets were analyzed in this study. Discovery cohort and duplication cohort (GSE33463 and GSE48149) were downloaded from GEO (http://www.ncbi.nlm.nih.gov/geo/).
